# Chronic Cancer-Related Pain in Children: A Narrative Review of Multimodal and Family-Centered Palliative Care Approach

**DOI:** 10.3390/children13050618

**Published:** 2026-04-29

**Authors:** Ada Maria Carstea, Alexandra Borda, Raluca Morosan, Adriana Elena Pittner, Estera Boeriu, Cristina Ionasiu Rebreanu, Stanciu-Lelcu Theia, Vulcanescu Dan Dumitru, Maria Mirabela Mihailescu Marin

**Affiliations:** 1 Department of Pediatrics II, “Louis Țurcanu” Emergency Clinical Hospital for Children, 300011 Timișoara, Romania; carstea.ada@umft.ro (A.M.C.); alexandra.borda@umft.ro (A.B.); raluca.morosan@umft.ro (R.M.); adriannepit@yahoo.com (A.E.P.); 2 Department XI—Pediatrics, “Victor Babeș” University of Medicine and Pharmacy, 300041 Timișoara, Romania; 3 Doctoral School, “Victor Babeș” University of Medicine and Pharmacy, 300041 Timișoara, Romania; cristina.ionasiu-rebreanu@umft.ro; 4 Department of Pediatrics III, “Louis Țurcanu” Emergency Clinical Hospital for Children, 300011 Timișoara, Romania; lelcu.theia@umft.ro; 5 Department III Functional Sciences, Chair of Pathophysiology, “Victor Babeș” University of Medicine and Pharmacy, 300041 Timișoara, Romania; 6 Centre for Translational Research and Systems Medicine, “Victor Babeș” University of Medicine and Pharmacy, 300041 Timișoara, Romania; 7 Department of Microbiology, “Victor Babeș” University of Medicine and Pharmacy, 300041 Timișoara, Romania; dan.vulcanescu@umft.ro; 8 Multidisciplinary Research Center on Antimicrobial Resistance (MULTI-REZ), Department of Microbiology, “Victor Babeș” University of Medicine and Pharmacy, 300041 Timișoara, Romania; 9 Research Department, “Louis Țurcanu” Emergency Clinical Hospital for Children, 300011 Timișoara, Romania; 10 Department of Medical and Surgical Specialties, Faculty of Medicine, Transilvania University of Brașov, 500036 Brașov, Romania; maria.marin@unitbv.ro; 11 Hospice Casa Speranței Foundation, 500074 Brașov, Romania

**Keywords:** chronic pain, children with cancer, pediatric oncology, pediatric palliative care, pain management, psychosocial support, family-centered care

## Abstract

**Highlights:**

**What are the main findings?**

- Chronic pain in children with cancer is a multidimensional experience shaped by disease- and treatment-related factors, as well as psychological, developmental, and family influences.
- Effective management requires multimodal care combining pharmacological treatment with psychological, supportive, and non-pharmacological interventions within a multidisciplinary framework.

**What are the implications of the main findings?**

- Early integration of pediatric palliative care and regular age-appropriate pain assessment may improve symptom control, quality of life, and family well-being.
- Family-centered, child-centered, and multidisciplinary strategies should be prioritized in clinical practice, while further pediatric-specific research is needed to strengthen the evidence base.

**Abstract:**

**Background**: Chronic pain in children with cancer is a major challenge in pediatric palliative care. It results from the interaction of disease-related and treatment-related factors, psychological distress, and the child’s family and social environment. When poorly controlled, it can impair quality of life, emotional development, social functioning, and family well-being. This narrative review examines the challenges and management strategies for chronic pain in children with cancer from a pediatric palliative care perspective, with attention to pain mechanisms, assessment difficulties, and psycho-emotional influences. **Methods**: This narrative review was based on a structured literature search conducted in PubMed/MEDLINE, Scopus, and Web of Science for English-language articles published between January 2000 and October 2025. Of 135 records identified, 15 studies judged most relevant to the thematic scope of the review were included in the final synthesis. A PRISMA-based flowchart was used to illustrate study identification and selection without implying a formal systematic review. **Results**: Chronic pain in children with cancer emerged as a multidimensional problem requiring an integrated approach to assessment and management, and some studies suggest that 20–26% of childhood cancer survivors experience persistent pain. Pharmacological strategies, including opioids and adjuvant medications, remain central, while psychological, supportive, and non-pharmacological interventions may complement multimodal care. **Conclusions**: Chronic pain in children with cancer should be managed through an integrated, individualized, and child-centered approach that addresses the physical, emotional, social, and relational dimensions of suffering and may improve quality of life for both children and their families.

## 1. Introduction

Childhood cancer remains a major global health burden, with recent epidemiological data confirming a substantial worldwide incidence across pediatric populations. Many of these children experience cancer-related pain that improves with oncologic treatment; however, some develop intractable pain as a consequence of disease progression or the sequelae of therapeutic interventions. Chronic pain in children with cancer represents a complex clinical problem with major implications for quality of life, psycho-emotional development, and overall prognosis, including in important subgroups such as children with hematologic malignancies. Unlike acute pain, chronic pain is persistent, multifactorial, and often insufficiently controlled, requiring an integrated, multidisciplinary approach [1,2].

Pediatric cancers encompass a broad spectrum of conditions, including leukemias, lymphomas, solid tumors, and central nervous system malignancies. These diseases are among the most important conditions encountered in pediatric oncology and are frequently accompanied by pain related to the underlying pathology, its complications, or the aggressive oncologic treatments administered [2].

Chronic pain is defined as pain that persists for more than three months or extends beyond the normal healing period. In the context of cancer, underlying mechanisms include tumor infiltration of bone or soft tissues, nerve compression, chronic inflammation, and adverse effects of chemotherapy and radiotherapy [3].

Children often experience difficulty in verbalizing pain, as pain perception is influenced by age, cognitive developmental stage, and family context. These factors complicate both the assessment and management of pain. Challenges in pediatric pain assessment arise from several factors, including the limited use in clinical practice of standardized age-appropriate assessment tools, underestimation of pain by healthcare professionals, the influence of anxiety and fear on pain expression, and the impact of cultural and familial factors on pain reporting [4,5].

Chronic pain in children with cancer must be understood as a complex phenomenon resulting from the interaction between biological mechanisms, psychological factors, and the social environment. According to the definition provided by the International Association for the Study of Pain, pain is “an unpleasant sensory and emotional experience associated with, or resembling that associated with, actual or potential tissue damage” [3]. In pediatric oncology, this definition is particularly relevant given the vulnerability of the child’s developmental process.

Pharmacological treatment of chronic pain in children with cancer generally follows established pediatric pain management principles, including age-adapted use of non-opioid analgesics, opioids, and adjuvant medications [1,5,6]. Non-opioid analgesics are used for mild pain; however, moderate to severe pain may require the use of opioids, with morphine remaining a reference analgesic in pediatric oncologic pain [6]. The use of opioids presents specific challenges, including accurate dose titration, monitoring of adverse effects, and family reluctance related to concerns about addiction. Available evidence suggests that controlled opioid administration in children can be safe in an appropriate therapeutic context, and that the risk of addiction appears low when these agents are carefully prescribed and monitored [5,7].

Adjuvant medications, including corticosteroids, anticonvulsants, and antidepressants, play an important role in the management of neuropathic or inflammatory pain associated with tumor infiltration and cytotoxic treatments [8].

Psycho-emotional pain is frequently underestimated in clinical practice, although it significantly influences the intensity of physical pain and therapeutic response. Children with oncological diseases have an increased risk of anxiety, depression, and adjustment disorders, factors that may amplify the pain experience [4]. Psychological interventions, particularly age-adapted cognitive–behavioral therapy, appear beneficial in reducing emotional distress and in developing coping mechanisms, thereby contributing to improved pain management [9]. Active family involvement is essential, as parental stress levels may be associated with the intensity of the child’s pain as reported by the child [10].

Although several recent publications have addressed specific aspects of pain in pediatric oncology, such as procedural or postoperative pain, survivorship-related pain, neuropathic pain, pain assessment tools, or broader pediatric palliative care models, a focused synthesis of chronic cancer-related pain in children from an integrated multimodal and family-centered palliative care perspective remains limited. In particular, the existing literature does not consistently bring together pain mechanisms, developmental and psychosocial influences, assessment challenges, and multidisciplinary management within a single clinically oriented framework. In this context, the present narrative review has two main objectives: first, to synthesize current evidence on the mechanisms and assessment of chronic cancer-related pain in children; and second, to evaluate multimodal management strategies, including pharmacological, interventional, psychological, and supportive approaches, within a family-centered pediatric palliative care framework.

## 2. Materials and Methods

This study was designed as a narrative review aimed at synthesizing the available literature on the epidemiology, assessment, and management of chronic pain in children with cancer within the context of pediatric palliative care. A narrative approach was considered appropriate as the available literature is heterogeneous in terms of study design, patient populations, and reported outcomes, making a thematic synthesis more suitable [3,11,12].

A structured literature search was conducted in PubMed/MEDLINE, Scopus, and Web of Science for English-language publications published between January 2000 and October 2025. The search combined controlled vocabulary and free-text terms related to chronic pain, pediatric oncology, cancer, palliative care, pain management, opioid therapy, neuropathic pain, psychosocial support, and multimodal analgesia, using Boolean operators such as AND and OR. Representative search combinations included (“chronic pain” AND “children with cancer”), (“pediatric oncology” AND “pain management”), (“palliative care” AND “cancer pain” AND child), and (“neuropathic pain” AND “pediatric cancer”).

Additional relevant publications were identified through targeted searches and manual screening of reference lists from selected articles and review papers. Publications were considered eligible when they addressed chronic cancer-related pain, pain assessment, pharmacological or multimodal pain management, psychosocial support, or family-centered care in children or adolescents with cancer. Original studies, reviews, clinical guidance papers, and selected contextually relevant supportive-care publications were considered. Publications focused predominantly on adult populations or exclusively on acute procedural pain were not prioritized, unless they offered useful contextual relevance for multimodal pain care in pediatric oncology.

**Figure 1:**
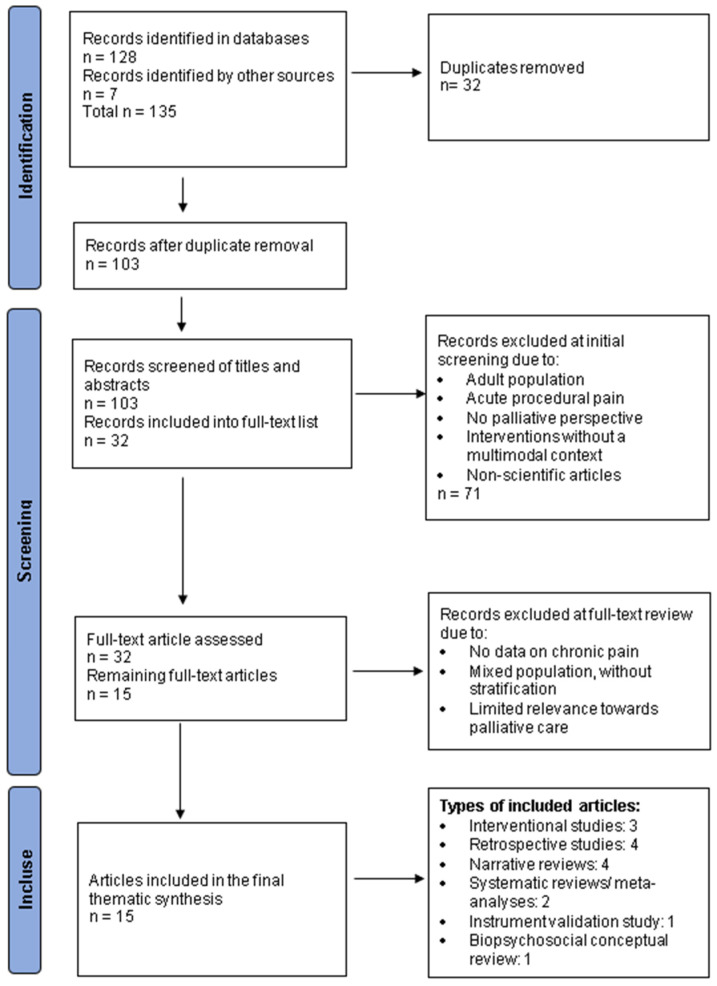
PRISMA-based visual aid for the study selection process.

A total of 135 articles were screened, of which 15 were retained as the most relevant for the thematic synthesis presented in this review and are presented in Table 1. The flowchart shown in Figure 1 is used as a PRISMA-based visual aid to illustrate the process of literature identification and thematic selection. This level of selectivity reflects the narrative nature of the review and the intention to prioritize studies most directly addressing chronic pain, pain assessment, multimodal management, psychosocial support, and family-centered care in children with cancer. Many identified records were excluded because they focused primarily on acute procedural pain, adult populations, or broader oncology topics with only indirect relevance to the present review.

The authors independently reviewed titles and abstracts and identified articles considered relevant to the aims of the review. Selected full texts were then examined in greater detail by A.E.P. and independently considered by the other two authors (E.B. and M.M.M.M.). Any differences in interpretation or selection were discussed and resolved by consensus.

The selected literature was organized thematically into the following domains: mechanisms and assessment of chronic pain, pharmacological management, non-pharmacological and psychological interventions, and multidisciplinary family-centered care. Given the heterogeneity of the available literature, the findings were synthesized qualitatively in a narrative manner.

## 3. Results

### 3.1. Overview of the Burden and Multidimensional Nature of Chronic Pain in Pediatric Oncology

Pain is one of the most frequent and debilitating symptoms in pediatric oncology, affecting both children undergoing active treatment and long-term cancer survivors. Recent literature suggests not only the high prevalence of pain in this context but also the need to explore innovative strategies for pain relief, including the integration of non-pharmacological interventions such as physical exercise as a complementary approach in the management of chronic pain in children and adolescents with cancer [13].

Recent evidence also supports the multidimensional nature of chronic pain in pediatric oncology. Chronic pain is not limited to nociceptive pain or treatment-related procedural pain, but may persist long after treatment completion, with important consequences for quality of life and for physical, emotional, and social functioning [13,25]. Psychological and contextual factors, including emotional distress, disruption of normal psychosocial developmental stages, and unmet needs affecting both the child and family, appear to contribute to the intensity and persistence of pain [15,16]. Several studies suggest that approximately 20–26% of childhood cancer survivors report chronic pain, with psychosocial factors such as post-traumatic stress symptoms and pain catastrophizing potentially influencing both pain persistence and severity [13,15,26].

The findings synthesized in this review suggest a shift beyond a strictly symptom-oriented approach toward a more integrated, child-centered model of pain management. Within pediatric oncology, chronic pain may be better understood not only as a physical symptom, but also as a multidimensional experience shaped by biological, psychological, developmental, and social factors. This interpretation is broadly consistent with prior review-level literature, such as materials published by Schulte et al. [27] or Papini et al. [28], which similarly identifies persistent pain as a multidimensional survivorship problem associated with functional impairment and reduced quality of life, despite the heterogeneity of the underlying evidence.

### 3.2. Neuropathic Mechanisms and Pain Assessment

Neuropathic mechanisms appear to contribute to chronic pain in pediatric cancer and are frequently associated with the administration of certain chemotherapeutic agents, particularly vincristine [14]. Recent studies have also suggested the potential impact of therapeutic combinations on the development of chemotherapy-induced neuropathy and associated pain [16,29]. In this context, gabapentinoids are frequently used in pediatric clinical practice, despite the absence of standardized evidence-based protocols, underscoring the need for analgesic strategies more specifically oriented toward the mechanisms underlying chronic oncologic pain in children [3].

Clinical cohort evaluations suggest that neuropathy may represent a significant component of chronic pain in pediatric oncology. Approximately 16% of pediatric cancer patients may present neuropathic pain, often secondary to nerve compression or chemotherapeutic treatment, including vincristine [14]. Among survivors, neuropathy may remain a contributor to chronic pain and has been associated with higher levels of pain catastrophizing [30,31]. This may be more relevant in some subgroups, including children with hematologic malignancies, in whom intensive treatment regimens may increase the burden of treatment-related neuropathic symptoms.

Recent work from 2025 has also focused on improving pain assessment. Hirata M. et al. described instruments designed for pain mapping in children with cancer, which may be useful for identifying and comparing clinical assessment measures relevant to practice [17]. In parallel, a recent review published in Cancers addressing the recognition and treatment of neuropathic pain in children and adolescents with cancer highlights the limited availability of robust clinical data and outlines current therapeutic directions [18].

A recurrent challenge identified in the literature is the under-recognition and undertreatment of chronic pain in pediatric oncology. Difficulties in pain assessment, particularly among younger children or those with communication barriers, may significantly contribute to this problem. Even when validated assessment tools are available, pain may still be underestimated, leading to delayed or insufficient intervention. These findings further support the importance of regular and age-appropriate pain assessment as an integral part of routine care. These findings further support the importance of regular and age-appropriate pain assessment as an integral part of routine care. This interpretation is broadly consistent with recent review-level literature, which likewise emphasizes both the limited pediatric-specific evidence for neuropathic pain management and the continuing lack of standardized assessment approaches for treatment-related neuropathy in children with cancer [18].

### 3.3. Pharmacological and Multimodal Pain Management

Pain management in pediatric oncology frequently involves pharmacological and multimodal approaches. Clinical studies suggest that combinations of non-opioid analgesics, opioids, and adjuvant medications such as gabapentin may be beneficial when carefully monitored, with possible reductions in pain intensity and improvements in quality of life. In this context, recent recommendations have suggested the inclusion of psychotherapy, physical exercise, and, in selected cases, interventional techniques as part of a broader multimodal strategy [19].

Interventional procedures for pain management, such as nerve blocks or epidural anesthesia, remain underutilized in pediatric clinical settings, although some reports suggest they may be helpful in managing pain that is refractory to standard treatment [20]. While these strategies remain insufficiently studied in children, they may represent therapeutic options for selected refractory cases, particularly when incorporated into a multidisciplinary treatment plan. Their earlier integration may also help reduce reliance on strong opioids and contribute to broader symptom control [21].

Opioids remain an important component in the treatment of severe cancer-related pain; however, the current literature generally favors their use in combination with other therapeutic modalities in order to reduce cumulative doses and adverse effects. Available studies suggest that opioid therapy combined with adjuvant agents, such as tricyclic antidepressants or gabapentinoids, may improve analgesic response in some cases of complex neuropathic pain [14].

A recent narrative review by Hall et al., examining the role of opioid analgesia and multimodal strategies in procedural and postoperative pain among pediatric cancer patients, also discusses opioid-sparing approaches. Techniques aimed at reducing opioid requirements, such as regional anesthesia, non-opioid adjuvants, combinations of opioids with sedatives, or ketamine-based regimens, were described as potentially comparable or, in some settings, possibly favorable relative to opioid-only strategies [22]. However, because this literature is oriented mainly toward procedural and postoperative pain, it should be interpreted here primarily as contextual evidence relevant to multimodal pain care rather than as direct evidence for chronic pain management.

A critical literature review conducted by Fuller et al. between 2022–2023 highlights the possible contribution of pharmacological, complementary, and procedure-related approaches within an integrated treatment plan, while also identifying priorities for future research [1]. Similarly, a retrospective study conducted by Bakır et al. in 2023, including 90 pediatric cancer patients under 18 years of age, reported that multimodal analgesic strategies were associated with reductions in pain intensity and appeared feasible and safe in this age group [19].

Pharmacological interventions remain central to pain control in pediatric oncology. However, the available literature suggests that their greatest clinical value may lie within a broader multimodal framework that also incorporates psychological, rehabilitative, and supportive strategies, in keeping with the goals of pediatric palliative care. This idea was also noted by Le-Short et al. [32].

### 3.4. Non-Pharmacological and Supportive Interventions

Non-pharmacological and complementary strategies are receiving increasing attention as adjunctive modalities with the potential to improve functional outcomes and reduce reliance on opioids. Although high-quality evidence remains limited, these interventions appear broadly consistent with the multidisciplinary philosophy of pediatric palliative care, which seeks to address total suffering rather than isolated physical symptoms [33].

Interventions such as psychological support, rehabilitation, adapted physical exercise, movement and dance therapy, and virtual reality have been reported to show promising results in improving coping, reducing pain, and enhancing function and quality of life in pediatric oncology patients [14,15,16,22]. Among these, psychological support, rehabilitation, and adapted exercise appear to have a relatively more consistent supportive role within multimodal care, whereas virtual reality and movement-based therapies remain promising but more exploratory in this context. Although reported benefits include improved physical functioning, reduced disability, and possibly lower long-term medication requirements [16,34], the available evidence remains heterogeneous, and their specific effect on chronic cancer-related pain in children is not yet firmly established.

Emerging evidence suggests that some non-pharmacological interventions, including virtual reality–based therapies, may help reduce procedural pain in pediatric oncology patients. Erdős et al. reported beneficial effects of non-pharmacological therapies, including virtual reality interventions [15], while Gürcan et al., in a more recent meta-analysis involving 494 participants, reported differences favoring digital techniques over traditional distraction methods [20]. However, these findings relate mainly to procedural pain and should therefore be interpreted cautiously in the context of chronic cancer-related pain. Virtual reality may represent a promising complementary approach within multimodal pain care, although its specific role in chronic pain management in children with cancer remains to be further clarified.

Overall, the findings suggest that supportive and non-pharmacological strategies may complement standard pain treatment when incorporated into a broader multidisciplinary plan. This interpretation is broadly consistent with recent review-level literature, which also suggests potential benefit from non-pharmacological interventions while emphasizing substantial heterogeneity in study design, clinical setting, and outcome measurement. Accordingly, the specific contribution of these interventions to chronic pain management in children with cancer still requires further clarification [35].

### 3.5. Psychosocial and Family-Centered Dimensions of Pain

The literature generally supports integrated pain management plans that combine pharmacological treatment with non-pharmacological methods, psychological support, physical rehabilitation, and family education in order to optimize pain control and improve quality of life [11]. The psychosocial dimensions of pain appear central to understanding and managing chronic pain in children with cancer. Pain persists not only as a physical sensation but also as an emotional and functional problem, affecting daily activities, sleep, and social relationships. Psychological interventions, cognitive–behavioral strategies, and family support are recognized as important components that may help reduce pain-related distress [21]. This interpretation is broadly consistent with recent review-level literature emphasizing the importance of psychosocial care in pediatric oncology, although some more recent pooled analyses suggest that measurable benefits on broader quality-of-life outcomes may be variable and may depend on intervention duration and study design [36].

An important theme emerging from the analyzed studies concerns the persistence of pain after completion of oncologic treatment. Up to 26% of pediatric cancer survivors report chronic pain, with substantial effects on functioning and quality of life and with associations reported for biopsychosocial factors such as post-traumatic stress symptoms and pain catastrophizing. These findings support the need for continued screening for chronic pain during long-term follow-up of childhood cancer survivors [23].

The psychological and emotional burden associated with malignant disease and its treatment appears to contribute importantly in pain perception and expression. Anxiety, fear, anticipatory distress, and depressive symptoms may amplify the pain experience, contributing to a bidirectional relationship between physical and emotional suffering [10]. Appropriate recognition of these factors is important for comprehensive care and supports the inclusion of psychotherapy, psychoeducation, music therapy, hypnosis, and, when appropriate, psychotropic medication alongside conventional treatment [33,37,38].

The literature also suggests that parental distress and parental coping styles may influence children’s pain behaviors and reported pain intensity, highlighting the role of the family both as a potential source of vulnerability and as a therapeutic resource [25]. Family-centered care therefore appears to represent an important strategy in reducing total pain. Educating parents about pain mechanisms, the use of analgesics, and non-pharmacological strategies may strengthen trust, reduce fears, particularly regarding opioid use, and improve adherence to therapeutic plans. In pediatric palliative care, empowering families represents not only an ethical responsibility but also a potentially meaningful clinical benefit. This broader emphasis is supported by recent review-level literature suggesting potential benefits of family psychosocial interventions in pediatric oncology, while also indicating that the evidence remains methodologically limited and not yet consistent across outcomes [39].

### 3.6. Clinical Implications and Current Gaps in the Literature

The results synthesized in this review suggest the need to move beyond a strictly symptom-oriented approach toward an integrated, child-centered model of pain management. The findings also support the early integration of pediatric palliative care alongside disease-directed treatment in children with cancer, including important subgroups such as those with hematologic malignancies. Multidisciplinary teams involving oncologists, palliative care specialists, psychologists, nurses, rehabilitation professionals, and other healthcare providers may be better positioned to address the complex interaction between physical symptoms and psychosocial distress. Such teams may also support specialized rehabilitation programs across the disease trajectory, from diagnosis to survivorship or end-of-life care [24].

This integrated approach appears to be associated with improved symptom control, improved communication, and better quality of life for both patients and families. At the same time, access to specialized pediatric palliative care services remains uneven across healthcare systems. Recent literature likewise supports early integration of pediatric palliative care in oncology, while also emphasizing that organizational barriers, limited resources, persistent misconceptions that associate palliative care exclusively with end-of-life care, and uneven service availability may delay referral and contribute to ongoing unmanaged pain and distress across healthcare systems [40].

The applicability of current evidence is further influenced by the marked heterogeneity of pediatric oncology populations. Differences in age, developmental stage, cancer type, disease status, and treatment regimens may affect pain mechanisms, the child’s ability to communicate pain, the choice of assessment tools, and the response to supportive or analgesic interventions. These considerations reinforce the importance of individualized, developmentally appropriate, and clinically context-sensitive pain management strategies.

The available literature also highlights several important gaps. High-quality studies specifically focused on chronic pain in children with cancer remain limited, and much of the evidence is derived from observational studies, mixed pediatric oncology populations, procedural-pain literature, or extrapolation from adult data. Robust data regarding the long-term outcomes of integrated pain management strategies in children are also lacking.

Future research should prioritize the development and validation of age-appropriate pain assessment tools, the evaluation of combined pharmacological and psychosocial interventions, and the exploration of family-centered and culturally sensitive models of care. Greater inclusion of patient- and family-reported outcomes is also needed to ensure that interventions reflect the lived experiences of children receiving oncologic and palliative care.

### 3.7. Strengths, Limitations and Future Perspectives

This narrative review is strengthened by its holistic perspective, integrating the biomedical, psychological, and social dimensions of chronic pain within a pediatric palliative care framework. This approach allows pain to be considered as a multidimensional experience and supports the inclusion of pharmacological, psychosocial, rehabilitative, and supportive care perspectives relevant to children with cancer.

At the same time, several limitations should be acknowledged. The available literature is heterogeneous in terms of study design, patient populations, interventions, and reported outcomes, which limits direct comparison across studies. Much of the evidence is observational or derived from mixed pediatric oncology cohorts, and some procedure-related pain studies were included mainly for contextual relevance within multimodal care. In addition, as a narrative review, the synthesis is inherently interpretive and may be influenced by selection and framing decisions. Moreover, no formal risk-of-bias assessment was performed. The marked heterogeneity of pediatric oncology populations with respect to age, developmental stage, cancer type, and treatment exposure may also limit the direct generalizability of the reviewed findings across all clinical contexts.

Future research should aim to better characterize chronic pain phenotypes in children with cancer, including neuropathic, treatment-related, and survivorship-associated pain patterns. More prospective pediatric studies are needed to evaluate integrated pain management models and to support more systematic and methodologically rigorous approaches using standardized outcomes, clearer pain definitions, and patient- and family-reported measures. Future evidence syntheses would also benefit from systematic reviews and, where feasible, meta-analyses focused on more homogeneous pediatric oncology subgroups, pain phenotypes, and treatment contexts. Greater attention to long-term functioning, quality of life, and culturally sensitive, developmentally appropriate care models may further improve the relevance of future research and its translation into clinical practice.

## 4. Conclusions

Chronic pain in children with cancer remains an important and often insufficiently addressed source of suffering in pediatric oncology. This narrative review suggests that pain management should extend beyond pharmacological symptom control to include the physical, psychological, social, and relational dimensions of pain. An integrated, child-centered, and family-oriented approach, supported by multidisciplinary teams, appears important for reducing total pain and improving quality of life for children and their families.

Further progress will likely depend on improving age-appropriate pain assessment, strengthening the evidence base for combined pharmacological and psychosocial interventions, and expanding access to specialized pediatric palliative care. Future research should prioritize prospective pediatric studies, standardized outcome measures, clearer characterization of chronic pain phenotypes, and greater inclusion of patient- and family-reported outcomes.

## Figures and Tables

**Table 1:** Synthesis of studies on pain in pediatric oncology included in the present review.

No.	Author	Year	No. of Patients	Study Population	Assessment Instruments	Article Type	Key Findings
1.	Fuller C et al. [1]	2022–2023	NA	Children with cancer	Pediatric pain scales	Review	Integrated pharmacological and psychosocial approaches are recommended.
2.	Dupuis LL et al. [4]	2021	243	Pediatric oncology patients	ESAS–Total Care	Validation study	Valid tool for assessing pain and total suffering.
3.	Rheel E et al. [9]	2022	NA	Children with cancer and survivors	Multidimensional instruments, Pain Science Education (PSE)	Review	Pain education reduces pain catastrophizing.
4.	Caru M et al. [13]	2023	NA	Children and adolescents with cancer	VAS, NRS	Review	Physical exercise reduces chronic pain.
5.	Soriano D et al. [14]	2024	66	Children with cancer	CTCAE, neuropathy scores	Observational study	Chemotherapy-associated peripheral neuropathy is a significant complication.
6.	Erdős S et al. [15]	2023	29	Pediatric oncology patients	Psychological and physiological variables	Pilot cross-over study	VR was associated with improved positive affect.
7.	Bryl K et al. [16]	2023	85	Children with cancer and their caregivers	Clinical observation	Retrospective study	Movement-based therapy improves emotional well-being.
8.	Hirata M et al. [17]	2025	NA	Infants, children, and adolescents with cancer	Pain assessment instruments	Scoping review protocol	Mapping of pediatric pain assessment tools.
9.	Coluzzi F et al. [18]	2025	NA	Children and adolescents with cancer	Neuropathy scales, NRS	Review	Neuropathic pain management is complex.
10.	Bakır M et al. [19]	2023	NR	Children with cancer	Pain scales	Retrospective study	Multimodal analgesia is effective and safe.
11.	Gürcan M et al. [20]	2025	494	Children with cancer	Validated pain scales	Meta-analysis	Non-pharmacological interventions reduce pain intensity.
12.	Fisher E et al. [21]	2018	NA	Pediatric oncology patients with chronic pain	Multidimensional scales	Review	The biopsychosocial model is essential for understanding and managing pain.
13.	Hall EA et al. [22]	2025	NA	Children with cancer	Procedural pain scales	Narrative review	Opioids remain essential for procedural pain management.
14.	Di Domenico F et al. [23]	2024	NR	Pediatric cancer survivors	QoL scales, NRS	Systematic review	Persistent pain negatively affects quality of life.
15.	L’Hotta AJ et al. [24]	2020	56	Children with cancer	Functional scales	Interventional study	Physical rehabilitation reduces disability.

NR = Not Reported. NA = Not Applicable. VAS = Visual Analog Scale. NRS = Numeric Rating Scale. CTCAE = Common Terminology Criteria for Adverse Effects. ESAS = Edmonton Symptom Assessment System. QoL = Quality of life.

## Data Availability

Not applicable.
